# Insulin Protects Cardiac Myocytes from Doxorubicin Toxicity by Sp1-Mediated Transactivation of Survivin

**DOI:** 10.1371/journal.pone.0135438

**Published:** 2015-08-13

**Authors:** Beom Seob Lee, Jaewon Oh, Sung Ku Kang, Sungha Park, Sang-Hak Lee, Donghoon Choi, Ji Hyung Chung, Youn Wook Chung, Seok-Min Kang

**Affiliations:** 1 Graduate Program in Science for Aging, Yonsei University, Seoul, Republic of Korea; 2 Cardiology Division, Severance Cardiovascular Hospital, Seoul, Republic of Korea; 3 Yonsei Cardiovascular Research Institute, Yonsei University College of Medicine, Seoul, Republic of Korea; 4 Severance Integrative Research Institute for Cerebral and Cardiovascular Diseases (SIRIC), Yonsei University Health System, Seoul, Republic of Korea; 5 Avon Old Farms School, Avon, Connecticut, United States of America; 6 Department of Applied Bioscience, College of Life Science, CHA University, Gyeonggi-do, Republic of Korea; University of Toronto, CANADA

## Abstract

Insulin inhibits ischemia/reperfusion-induced myocardial apoptosis through the PI3K/Akt/mTOR pathway. Survivin is a key regulator of anti-apoptosis against doxorubicin-induced cardiotoxicity. Insulin increases survivin expression in cardiac myocytes to mediate cytoprotection. However, the mechanism by which survivin mediates the protective effect of insulin against doxorubicin-associated injury remains to be determined. In this study, we demonstrated that pretreatment of H9c2 cardiac myocytes with insulin resulted in a significant decrease in doxorubicin-induced apoptotic cell death by reducing cytochrome c release and caspase-3 activation. Doxorubicin-induced reduction of survivin mRNA and protein levels was also significantly perturbed by insulin pretreatment. Reducing survivin expression with survivin siRNA abrogated insulin-mediated inhibition of caspase-3 activation, suggesting that insulin signals to survivin inhibited caspase-3 activation. Interestingly, pretreatment of H9c2 cells with insulin or MG132, a proteasome inhibitor, inhibited doxorubicin-induced degradation of the transcription factor Sp1. ChIP assay showed that pretreatment with insulin inhibited doxorubicin-stimulated Sp1 dissociation from the *survivin* promoter. Finally using pharmacological inhibitors of the PI3K pathway, we showed that insulin-mediated activation of the PI3K/Akt/mTORC1 pathway prevented doxorubicin-induced proteasome-mediated degradation of Sp1. Taken together, insulin pretreatment confers a protective effect against doxorubicin-induced cardiotoxicity by promoting Sp1-mediated transactivation of survivin to inhibit apoptosis. Our study is the first to define a role for survivin in cellular protection by insulin against doxorubicin-associated injury and show that Sp1 is a critical factor in the transcriptional regulation of survivin.

## Introduction

Survivin (encoded by Birc5), a member of the inhibitor of apoptosis protein (IAP) family, plays a crucial role in regulating apoptosis and contributes to tumor progression [1, 2]. Survivin suppresses mitochondrial apoptosis by inhibiting caspase-9 activities in concert with the caspase inhibitor, XIAP [3].

Expression of the *survivin* gene is largely regulated at the transcription level [4]. The *survivin* gene promoter region contains binding sites for numerous transcription factors, including NF-κB, GATA-1, Stat3, E2F, c-myc, KLF5, DEC1, Sp1, Sp3, HIF-1α and tumor suppressors p53 and Rb [1, 4–11]. Hoffman *et al*. reported that down-regulation of *survivin* transcription by the DNA-damaging agent doxorubicin is mediated by p53 induction [12]. Other works have shown that p53 suppresses *survivin* gene expression both directly and indirectly [4–6, 13, 14]. Conversely, it was demonstrated that Sp1 and Sp3 transcription factors transactivate the *survivin* promoter [15].

Accumulated evidences have suggested that survivin is cardioprotective [16–18]. In the spontaneously hypertensive rat, the *survivin* expression is inversely correlated with apoptosis and adverse cardiac remodeling [19]. Cardiac-specific deletion of survivin results in premature cardiac death due to a dramatic reduction in cardiac myocyte numbers [20]. In addition, survivin is associated with cardiac myocyte size and DNA content in the failing human heart [21].

Doxorubicin, a quinine-containing anthracycline anticancer drug, is a highly effective chemotherapeutic widely used against human hematological malignancies and solid tumors. Although it has a strong anticancer effect, doxorubicin is also known to cause cardiotoxicity that leads to hypotension, arrhythmia, depression of left ventricular function and heart failure [22, 23]. A variety of studies have suggested the mechanism involved in doxorubicin-induced cardiotoxicity and apoptosis, including reactive oxygen species (ROS) production, caspase activation and cell cycle arrest [24, 25]. The survivin gene therapy prevents myocytes from apoptosis and attenuates left ventricular systolic dysfunction in the doxorubicin-induced heart model [26]. Recently, we also reported the protective effect of survivin against doxorubicin-induced cell death in H9c2 cardiac myocytes [27]. The contribution of the phophatidylinositide-3-kinase (PI3K)/Akt/mammalian target of rapamycin (mTOR) axis to survivin expression is observed not only in various cancer cells [28, 29], but also in normal cells including cardiac myocytes [30]. In the latter case, survivin plays a critical role in the cardioprotection of insulin against myocardial ischemia/reperfusion (I/R) injury through the PI3K/Akt/mTOR signaling pathway. However the contribution of the PI3K/Akt/mTOR pathway and survivin in insulin-mediated protection of cardiac myocytes from doxorubicin-associated toxicity remains to be determined. In this study, we set out to elucidate the mechanism by which insulin signals to survivin to mediate cytoprotection against doxorubicin-associated injury in the H9c2 cardiac myocyte cell line.

## Materials and Methods

### Reagents and antibodies

Insulin, human recombinant from *Saccharomyses cerevisiae*, was purchased from Sigma-Aldrich. Doxorubicin was obtained from Tocris. Anti-survivin, anti-caspase-3 (cleaved form), anti-phospho-p53 (Ser^15^), anti-phospho-Akt (Ser^473^), anti-Akt, anti-phospho-mTOR (Ser^2481^) and anti-mTOR antibodies were obtained from Cell Signaling. Anti-Sp1, anti-phospho-p70S6K (Thr^421^/Ser^424^), anti-p70S6K, anti-β-actin, anti-GAPDH, anti-Smac/DIABLO antibodies and p70S6K inhibitor PF4708671 were purchased from Santa Cruz Biotechnology. Anti-VDAC1, anti-p53, anti-Bcl-2, and anti-BAX antibodies were obtained from Abcam. PI3K inhibitor LY294002 and mTOR inhibitor Rapamycin were purchased from Calbiochem. Anti-cytochrome c antibody was included in ApoAlert Cell Fractionation Kit (Clontech).

### Cell culture

The rat heart-derived myoblast cell line H9c2 (2–1) cardiac myocytes, was obtained from the American Type Culture Collection (ATCC CRL-1446). Cells were maintained in Dulbecco’s modified Eagle’s medium (DMEM) supplement with 10% fetal bovine serum (FBS) and 100 U/ml of penicillin and 100 μg/ml of streptomycin (Gibco) at 37°C in a humidified atmosphere with 5% CO_2_. All experiments were performed using cells between 15 to 25 passage numbers. After adaptation in DMEM containing 10% FBS for 24 h, cells were starved in DMEM containing 0.5% FBS for 24 h. After starvation, cells were pretreated with insulin in 0.5% FBS containing DMEM for 1 h prior to doxorubicin treatment for 24 h.

### Cell viability and cell death assay

Cell viability was measured by MTT assay and cell death was determined by TUNEL assay (Promega), cytochrome c release and caspase-3 activity assay (ApoAlert CPP32/caspase-3 assay kit, BD Biosciences), which were performed as described previously [27].

### Confocal immunofluorescence microscopy

Immunofluorescence microscopy was performed as previously reported [27]. Briefly, H9c2 cells cultured on Lab-Tek chamber slides (Nalgene Nunc) were fixed with 3% paraformaldehyde and permeablized with 0.5% Triton X-100. After blocking with PBS containing 0.3% goat serum and 5% bovine serum albumin, the slides were incubated with survivin antibody and mounted with ProLongantifade reagent containing DAPI. The immunoreactive signals were visualized by confocal laser scanning microscope LSM700 (Carl Zeiss, Germany).

### Subcellular fractionation and Immunoblot analysis

Mitochondrial and cytosolic fractions were obtained using Qproteome Cell Compartment Kit (Qiagen) according to the manufacturer's instructions, and protein sample preparation and immunoblot analysis were performed as described previously [27].

### Reverse transcription-polymerase chain reaction (RT-PCR)

Total RNA was isolated using QIAzol-Regent (Qiagen) and reverse transcribed using Omniscript Reverse Transcriptase (Qiagen). The cDNAs were amplified using TaKaRa Ex Taq polymerase (Takara). The sequences of the primers were as follows: survivin F, 5'-ATG GGT GCT ACG GCG CTG CCC-3'; survivin R, 5'-TCA GCG TAA GGC AGC CAG CTG-3'; Sp1 F, 5'- GGA GAA AAC AGC CCA GGA TGC-3'; Sp1 R, 5'-CTC ATC CGA ACG TGT GAA GC-3'; GAPDH F, 5'-AAT GCA TCC TGC ACC ACC AAC TGC-3'; GAPDH R, 5'-GGA GGC CAT GTA GGC CAT GAG GTC-3'. PCR products were separated by electrophoresis in a 1% agarose gel containing Gel-red (Biotium).

### RNA interference

H9c2 cells were transfected with scrambled RNA or siRNA targeted to either *survivin* or *Sp1* gene using Lipofectamine RNA iMAX (Invitrogen) according to the manufacturer’s protocol. *survivin* siRNA targeting sequences, 5'-GCA AAG GAG ACC AAC AAC AUU-3' and 5'-UGU UGU UGG UCU CCU UUG CUU-3’, and *Sp1* siRNA targeting sequences, 5'-AGC CUU GAA GUG UAG CUA UUU-3' and 5'-AUA GCU ACA CUU CAA GGC UUU-3' were synthesized by Genolution Pharmaceuticals. Scrambled RNA was purchased from Santa Cruz Biotechnology.

### Chromatin immunoprecipitation (ChIP)

ChIP assay was performed according to Hsu *et al*. with minor modifications [31]. Briefly, formaldehyde-treated nuclear lysates were subjected to immunoprecipitation with anti-Sp1 and anti-p53 antibodies. The cross-linked chromatin complex was reversed in the presence of proteinase K and DNA fragments were purified. The DNA fragment (257-bp) of *survivin* promoter region (between -265 and -9) was amplified by PCR using a pair of primers: Rat survivin promoter F, 5’-AGG ACA CAA CTC CCA GCA AG- 3’; Rat survivin promoter R, 5’-CGC CAC AAT CCC TAA TTC AA- 3’. PCR condition was as follows: at 95°C for 30 sec; at 56°C for 30 sec; and at 72°C for 60 sec. After 36 cycles of PCR, products were analyzed by 2% agarose gel electrophoresis. For input data (5%), 25 μl aliquots of 500 μl samples were taken before immunoprecipitation.

### Statistical analysis

Data were expressed as mean ±S.D. One-way ANOVA with Bonferroni post hoc correction was used for comparison between the groups using the Prism software (GraphPad Software Inc.). Values of *p* less than 0.05 were considered statistically significant.

## Results

### Insulin protects H9c2 cardiac myocytes from doxorubicin-induced cell death

**Fig 1:**
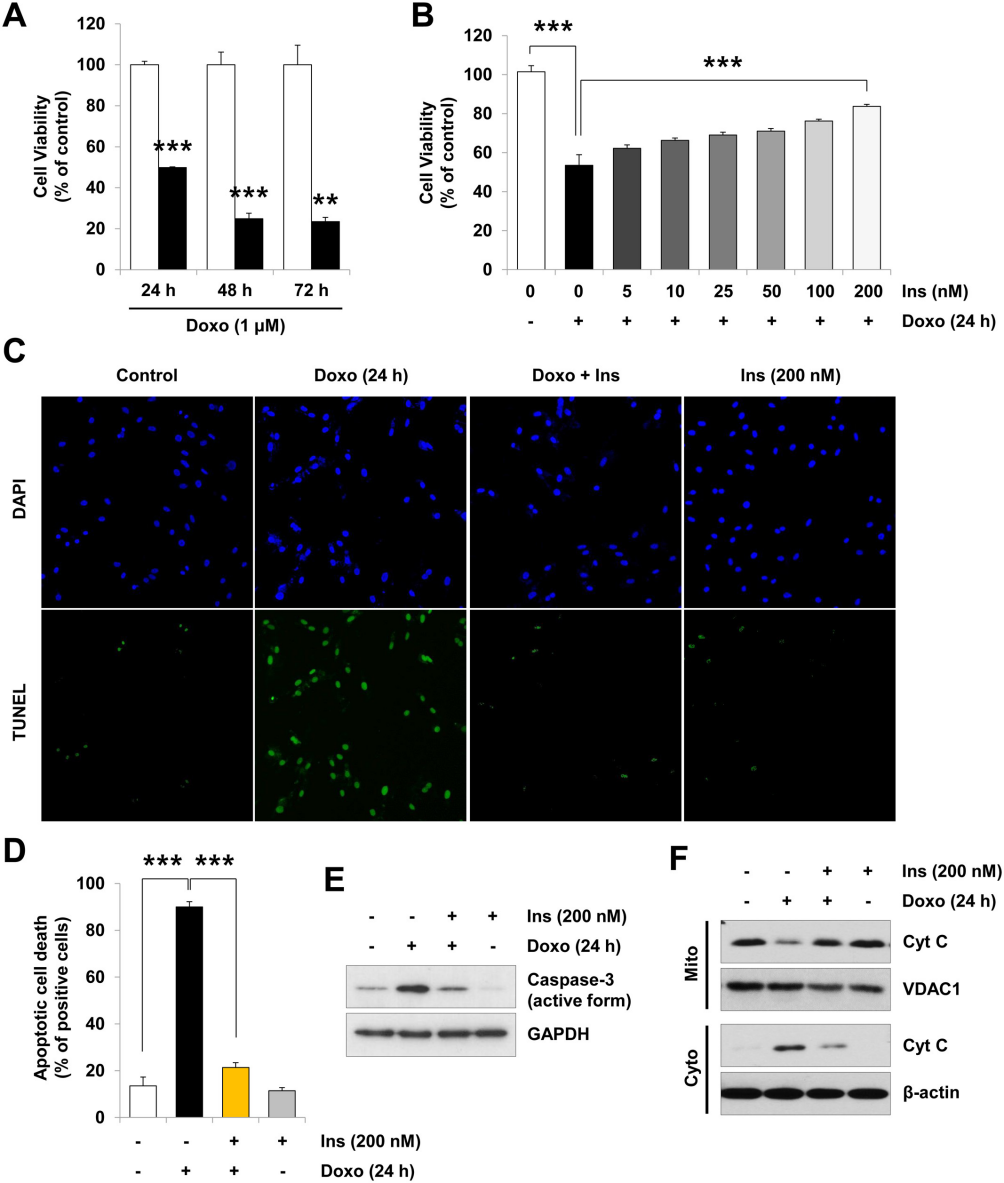
Protective effect of insulin on the doxorubicin-induced cell death in H9c2 cardiac myocytes. (A) H9c2 cardiac myocytes were left untreated or treated with 1 μM doxorubicin (*Doxo*) for 24 h, 48 h, and 72 h. (B) Serum-deprived H9c2 cardiac myocytes were left untreated or pretreated with the indicated concentration of insulin (*Ins*) for 1 h prior to treatment with 1 μM doxorubicin (*Doxo*) for 24 h. Cell viability was assessed by the MTT assay (**p < 0.01; ***p < 0.001, n = 3 performed in triplicates). (C–F) Serum-deprived cells were left untreated or pretreated with insulin (200 nM) for 1 h prior to treatment with doxorubicin (1 μM) for 24 h. (C) Represented images of the TUNEL assay (100×). Treated cells were incubated with TUNEL reaction mixture, followed by staining with DAPI. (D) The TUNEL-stained cells were counted under fluorescence microscopy and presented as a bar graph (***p < 0.001, n = 3 performed in triplicates). (E) Caspase-3 activity was determined by immunoblot analysis of active form of caspase-3. (F) Mitochondrial (*Mito*) and cytosolic (*Cyto*) fractions were separated by SDS-PAGE gel and analyzed by immunoblotting with anti-cytochrome C (*Cyt C*) antibodies. GAPDH or β-actin bands show that equal amounts of sample were loaded and VDAC1 is used as a loading control for mitochondrial fraction. Note that blots represent one of three independent experiments. Values are mean ±S.D.

To investigate whether insulin protects H9c2 cardiac myocytes against doxorubicin-induced injury, H9c2 cardiac myocytes were pretreated with insulin for 1 h prior to doxorubicin treatment. Consistent with previous reports [27], stimulation of H9c2 cells with doxorubicin for 24 h, 48 h or 72 h reduced cell viability to 49.8 ± 0.4%, 24.9 ± 2.7% and 23.5 ± 2.0%, respectively (Fig 1A). However, doxorubicin-stimulated cell death was significantly perturbed by pretreatment with 200 nM of insulin resulting in 84.2 ± 0.8% of H9c2 cell viability (Fig 1B). Consistently, doxorubicin treatment stimulated apoptosis in 90.0 ± 2.3% of H9c2 cells, and insulin pretreatment dramatically reduced the doxorubicin-induced apoptotic cell death to 21.4 ± 2.0%, as determined by TUNEL assay (Fig 1C and 1D). Similar to the previous findings [27], pretreatment with doxorubicin alone stimulated activation of other markers of apoptotic cell death in H9c2 cells, including activation of caspase-3, release of pro-apoptotic mitochondrial proteins cytochrome c and Smac/DIABLO to cytosol, decreasing anti-apoptotic Bcl-2 protein levels and increasing pro-apoptotic Bax protein levels. However, pretreatment with insulin prevented doxorubicin-stimulated caspase-3 activation (Fig 1E), release of cytochrome c (Fig 1F) and Smac/DIABLO (S1A Fig), decreased Bcl-2 protein levels and increased Bax protein levels (S1B Fig). These results suggest that insulin protects H9c2 cardiac myocytes from doxorubicin toxicity by blocking apoptosis.

### Insulin inhibits the doxorubicin-induced survivin down-regulation in H9c2 cardiac myocytes

**Fig 2:**
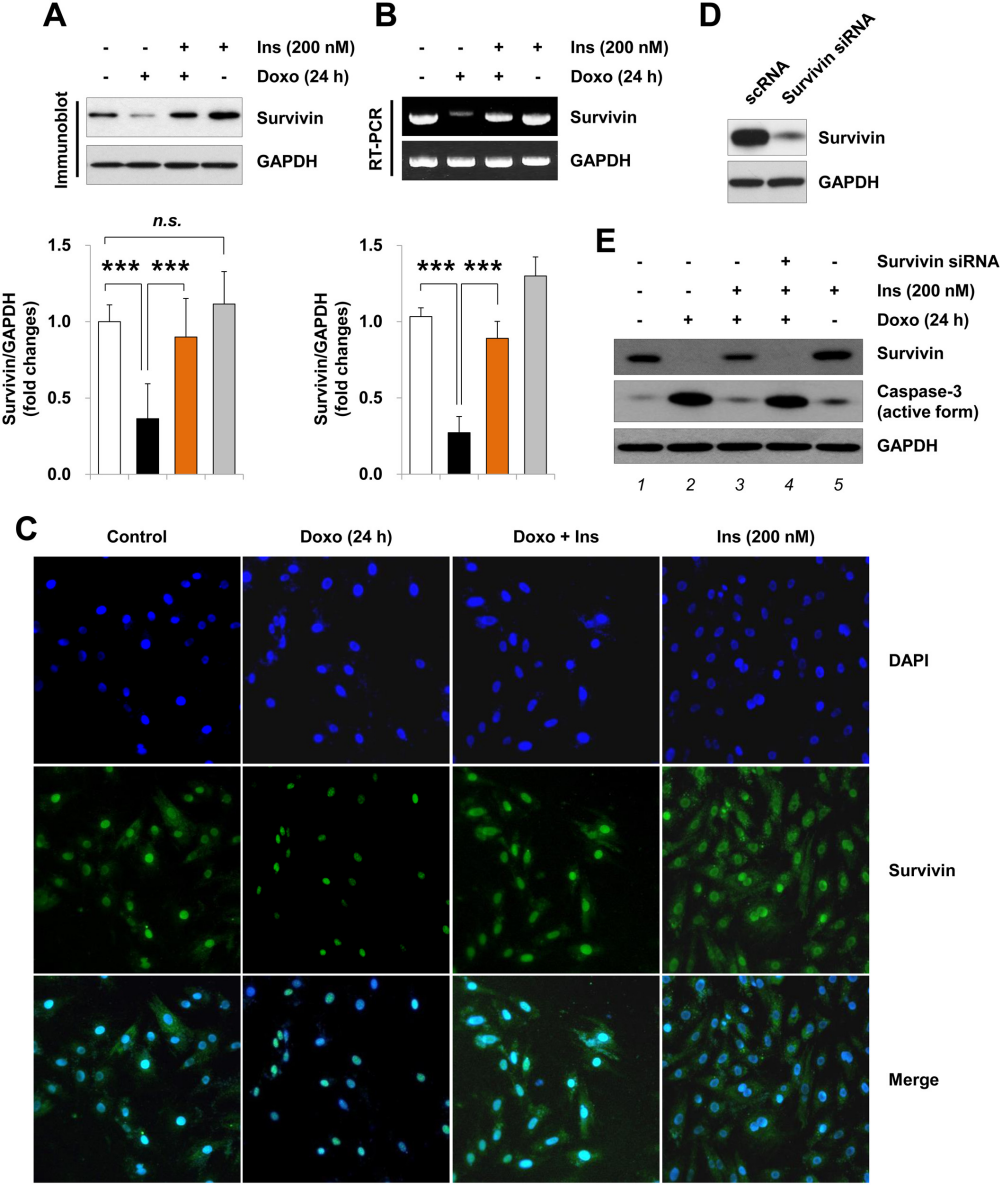
Inhibitory effect of insulin on the doxorubicin-induced survivin down-regulation. (A, B) Serum-deprived H9c2 cardiac myocytes were left untreated or pretreated with 200 nM insulin (*Ins*) for 1 h and treated with 1 μM doxorubicin (*Doxo*) for 24 h. (A) Whole cell lysates were separated by SDS-PAGE gel and analyzed by immunoblotting with anti-survivin antibody. Graph represents the mean ±S.D. of the normalized densitometric analysis of survivin protein levels from 8 independent experiments (***p < 0.001, n = 8). (B) Total RNA was analyzed by RT-PCR (28 cycles) using primers specific to *survivin* gene. Graph represents the mean ±S.D. of the normalized densitometric analysis of *survivin* mRNA levels from 3 independent experiments (***p < 0.001, n = 3). (C) Immunofluorescence microscopy images were obtained using anti-survivin antibody followed by staining with DAPI (200×). (D, E) One day after transfection with either scrambled RNA (scRNA) or survivin siRNA (20 nM), H9c2 cardiac myocytes were left untreated or pretreated with insulin (200 nM) for 1 h and treated with doxorubicin (1 μM) for 24 h. Whole cell lysates were blotted with antibodies against survivin and cleaved-caspase-3 (active form). Note that the blots in *panel D* and *E* represent one of three independent experiments. *n*.*s*. not significant.

Previously Si et al. showed that insulin inhibits I/R-induced myocardial apoptosis by stimulating the survival signaling cascade PI3K/Akt/mTOR/survivin pathway [30]. Further, survivin has been shown to be cardioprotective [16–18]. Thus, we examined whether survivin expression is altered by insulin stimuli in doxorubicin-associated injury. Insulin treatment alone did not affect survivin protein levels in H9c2 cells (*p* = 0.2, n = 8) (S2 Fig). Interestingly, H9c2 cells treated with doxorubicin had reduced levels of survivin protein (Fig 2A and 2C) and mRNA (Fig 2B), and these reductions were suppressed with insulin pretreatment. The role of survivin in insulin-mediated H9c2 cell protection was investigated using the siRNA knockdown method. H9c2 cardiac myocytes were transfected with the siRNA targeting *survivin* to reduce its expression. Fig 2D shows the efficiency of survivin siRNA in reducing the protein level of survivin. As shown in Fig 2E, the ability of insulin to suppress doxorubicin-mediated caspase-3 activation was completely perturbed by survivin knockdown (*lane 4*). These findings suggest that survivin plays a pivotal role in the cytoprotection of insulin by blocking the doxorubicin-induced apoptotic activity in H9c2 cardiac myocytes.

### Insulin inhibits doxorubicin-induced up-regulation of p53 and down-regulation of Sp1

**Fig 3:**
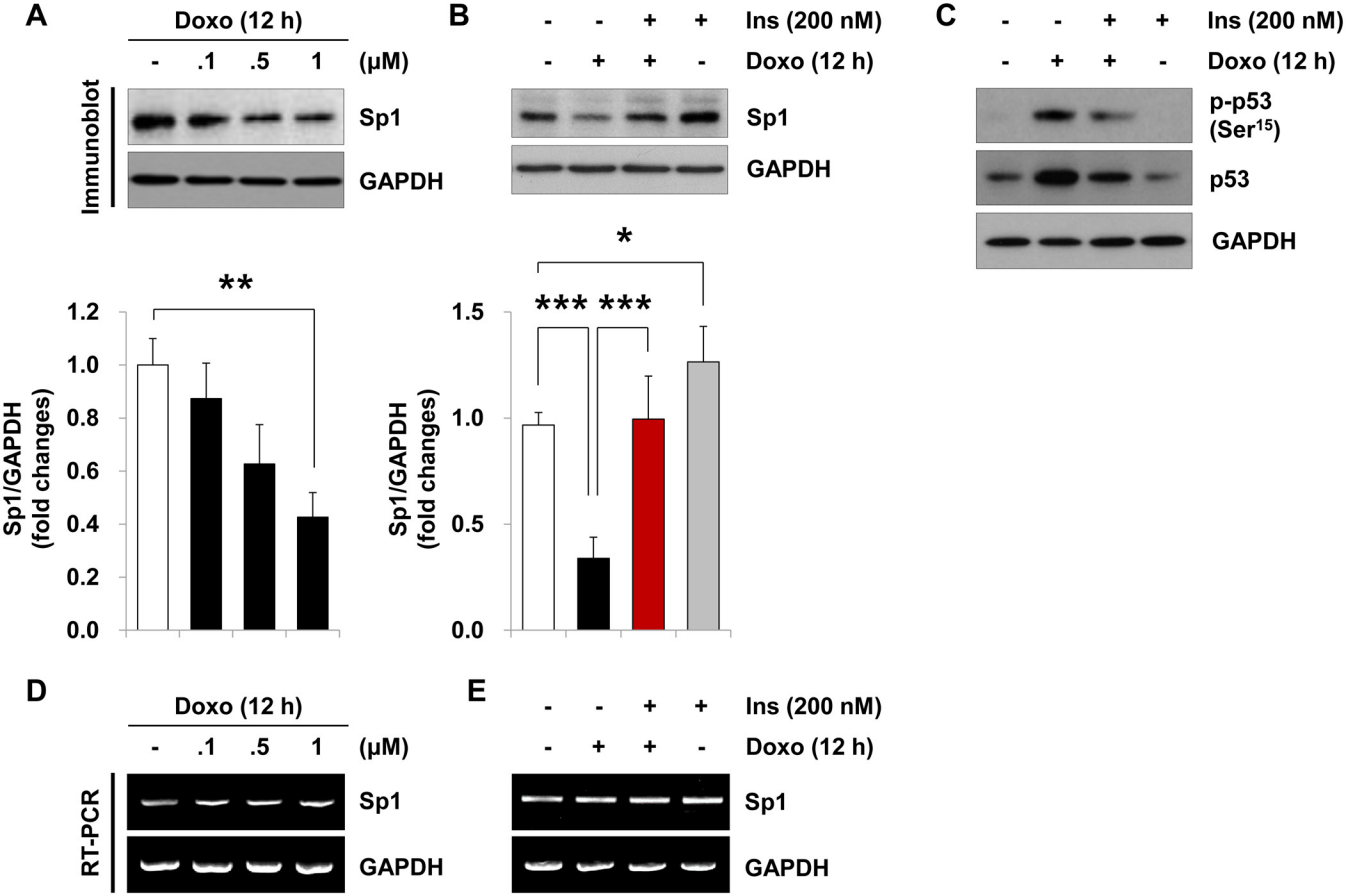
Effect of insulin on the doxorubicin-induced changes in Sp1 and p53. (A, D) H9c2 cardiac myocytes were left untreated or treated with the indicated concentration of doxorubicin (*Doxo*) for 12 h, and (B, C, E) serum-deprived cells were left untreated or pretreated with insulin (200 nM) for 1 h prior to treatment with doxorubicin (1 μM) for 12 h. Whole cell lysates were analyzed by immunoblotting with anti-Sp1, anti-phospho-p53 (Ser^15^), anti-p53 and anti-GAPDH antibodies. Graphs represent the mean ±S.D. of the normalized densitometric analyses of Sp1 protein levels (A, **p < 0.01, n = 3; B, ***p < 0.001, n = 5). (D, E) Sp1 mRNA amount was determined by RT-PCR (28 cycles). Note that the blots in *panel C* represent one of three independent experiments.

The *survivin* gene promoter contains several Sp1 and p53 binding sites with some variations between species, indicating the evolutionarily conserved participation of Sp1 and p53 in the *survivin* gene regulation. We found that doxorubicin treatment reduced Sp1 protein levels in time- (S3A Fig) and dose-dependent manners (Fig 3A). This correlates with doxorubicin-stimulated decrease in survivin mRNA levels at 12 h (S3B Fig), followed by a decrease in survivin protein levels at 18 h (S3A Fig) in response to doxorubicin treatment. Conversely, insulin treatment alone significantly increased Sp1 protein by 34.8% (Fig 3B). The reduction of Sp1 protein due to doxorubicin treatment was significantly prevented by pretreatment with insulin (Fig 3B). In contrast to its protein levels, the mRNA levels of Sp1 were unaffected by doxorubicin treatment with or without insulin pretreatment (Fig 3D, 3E and S4 Fig). Similar to the findings in various human cancer cell lines [12], doxorubicin treatment alone stimulated an increase in phosphorylation of p53 at Ser^15^ and total p53 levels (S3A Fig). Interestingly, insulin pretreatment diminished doxorubicin-stimulated increase in p53 phosphorylation at Ser^15^ and total p53 protein levels (Fig 3C).

### Doxorubicin-induced protein degradation of Sp1 is associated with proteasome-mediated proteolysis

**Fig 4:**
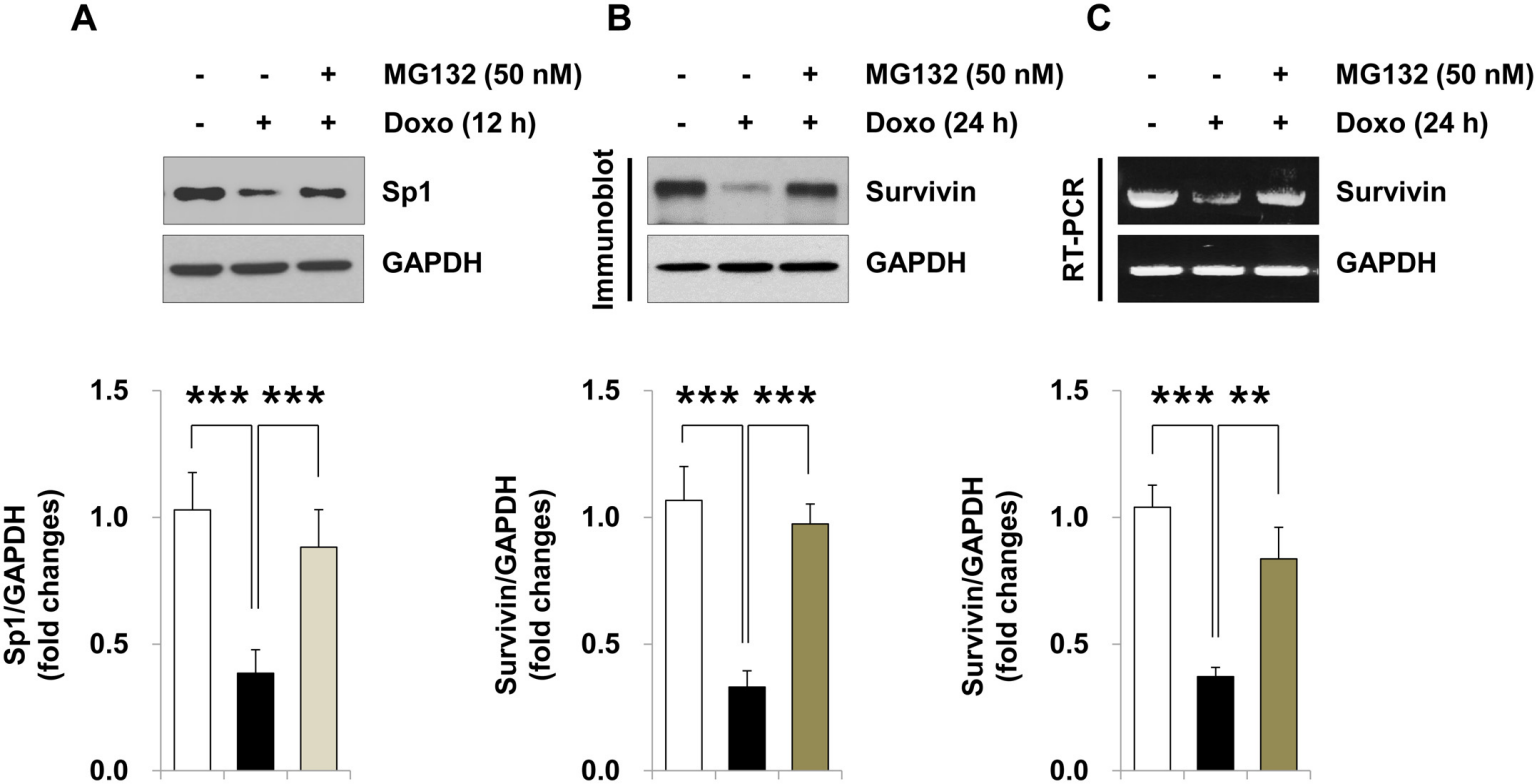
Doxorubicin-induced Sp1 degradation by proteasome-mediated proteolysis. (A–C) H9c2 cardiac myocytes were pretreated with proteasome inhibitor MG132 (50 nM) for 1 h and treated with 1 μM doxorubicin (*Doxo*) for either 12 h (A) or 24 h (B, C). (A, B) Whole cell lysates were analyzed by immunoblotting using anti-Sp1 and anti-survivin antibodies. Graphs represent the mean ±S.D. of the normalized densitometric analyses of Sp1 (A, n = 5) or survivin (B, n = 5) protein levels (***p < 0.001). (C) Total RNA was analyzed by RT-PCR (28 cycles) with specific primers to *survivin* gene. Graph represents the mean ±S.D. of the normalized densitometric analysis of *survivin* mRNA levels (**p < 0.01; ***p < 0.001, n = 3).

To test whether doxorubicin-stimulated decrease in Sp1 protein level relies on proteasome-dependent protein degradation, we pretreated H9c2 cells with the proteasome inhibitor MG132. Intriguingly, similar to insulin pretreatment (Fig 3B), the reduction of Sp1 protein levels due to doxorubicin treatment was significantly prevented by pretreatment with MG132 (Fig 4A). Consistently, MG132 pretreatment also inhibited the doxorubicin-mediated down-regulation of survivin in both protein and mRNA levels (Fig 4B and 4C, respectively). These data suggest that doxorubicin treatment triggers degradation of Sp1 protein via proteasome-mediated proteolysis to suppress survivin expression, and insulin pretreatment inhibits this process through an unknown mechanism.

### Insulin restores the transcriptional activity of Sp1

**Fig 5:**
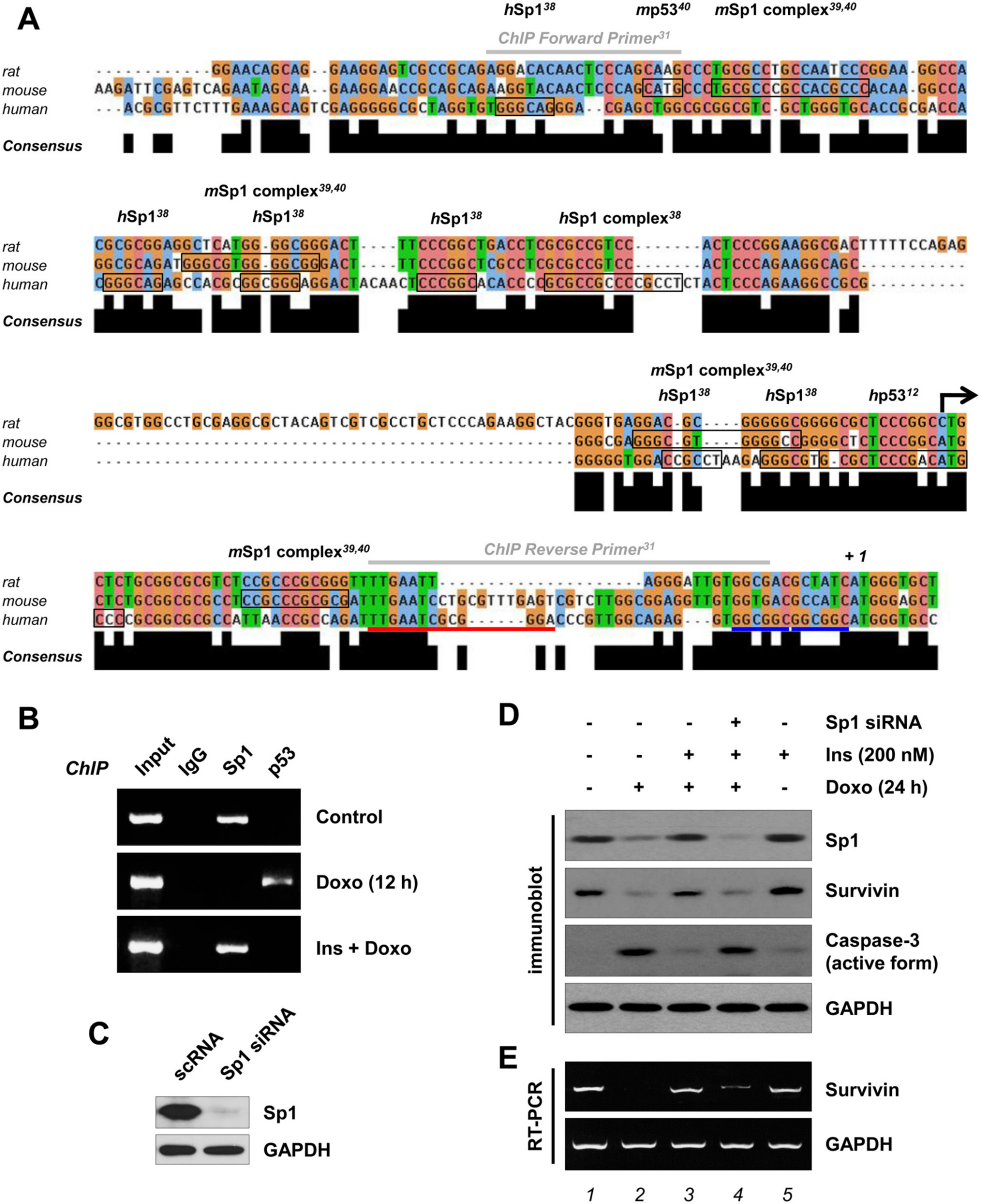
Effect of doxorubicin and/or insulin on the transcriptional activity of Sp1 and p53. (A) Multiple sequence alignment (ClustalW2) of the proximal promoter regions of the rat, mouse and human *survivin* genes. Black arrow indicates the transcriptional start site and +1 points out the translation start codon. Canonical p53, Sp1, Sp1-like sites of the mouse and human *survivin* genes are boxed [12, 38–40]. Two gray bars indicate the primers for ChIP analysis [31]; a red bar corresponds to human CHR sequences; two blue bars highlight human CDE regions [50]. (B) Serum-deprived cells were left untreated or pretreated with 200 nM insulin (*Ins*) for 1 h and treated with 1 μM doxorubicin (*Doxo*) for 12 h. Cross-linked cell lysates were subjected to ChIP analysis with anti-Sp1 or anti p53-antibody. RT-PCR (36 cycles) was performed with ChIP primers as listed in Materials and Methods (n = 5). (C–E) One day after transfection with Sp1 siRNA (20 nM), cells were left untreated or pretreated with insulin (200 nM) for 1 h and treated with doxorubicin (1 μM) for 24 h. Whole cell lysates were immunoblotted with anti-Sp1, anti-survivin and anti-caspase-3 (active form) antibodies (C, D), and total RNA was analyzed by RT-PCR (28 cycles) with specific primers to *survivin* gene (E). Note that these results represent one of three independent experiments.

To identify the Sp1 and p53 binding sites on the rat *survivin* promoter, we aligned the 5’-flanking region of the rat, mouse and human *survivin* gene (Fig 5A). Based on the alignment, we predicted a maximum of nine Sp1 and Sp1-like sites and two p53 binding sites in the promoter region (between -265 and -9) of the rat *survivin* gene. To determine whether pretreatment with insulin alters doxorubicin-stimulated binding of Sp1 and/or p53 to the *survivin* promoter, we performed ChIP experiments using H9c2 cardiac myocytes. The fixed cell extracts were incubated with anti-Sp1 or anti-p53 antibodies. The DNA-protein (promoter-transcription factor) complexes captured by the antibodies were amplified by PCR to detect the presence of the *survivin* promoter. If Sp1 or p53 is bound to the *survivin* promoter, anti-Sp1 or anti-p53 antibodies will be able to pull down the *survivin* promoter. Data presented in Fig 5B demonstrated that in the absence of both doxorubicin and insulin, the *survivin* promoter was occupied by Sp1, but not by p53. Following doxorubicin treatment of the H9c2 cardiac myocytes, the *survivin* promoter had reduced levels of bound Sp1, but had increased level of bound p53. This observation is in accordance with the findings of Esteve *et al*. using human HCT116 cell line (doxorubicin treatment) [13] and recently Hsu et al. using rat C6 glioma cell line (trichostatin A treatment) [31]. However, when H9c2 cardiac myocytes were pretreated with insulin prior to doxorubicin treatment, Sp1 binding to the *survivin* promoter was maintained while p53 binding to the *survivin* promoter was prevented by insulin.

To test whether Sp1 is a critical player in the insulin-induced survivin-mediated H9c2 cell protection, H9c2 cells were transfected with the Sp1 siRNA to reduce Sp1 expression. The effectiveness of the knockdown is shown in Fig 5C. As shown in Fig 5D and 5E, when Sp1 expression was reduced by siRNA, both *survivin* mRNA and its gene product, respectively, were substantially reduced. Similar to the effect of *survivin* knockdown (Fig 2E, *lane 4*), insulin-mediated suppression of doxorubicin-stimulated caspase-3 activation was also perturbed by reduction of Sp1 expression with Sp1 siRNA *(lane 4*). These evidences suggest that insulin protects H9c2 cardiac myocytes from doxorubicin-induced apoptosis by blocking the proteasome-mediated degradation of Sp1 resulting in Sp1-mediated transactivation of *survivin*.

### Insulin inhibits Sp1 degradation via activation of the PI3K/mTORC1/p70S6K pathway

**Fig 6:**
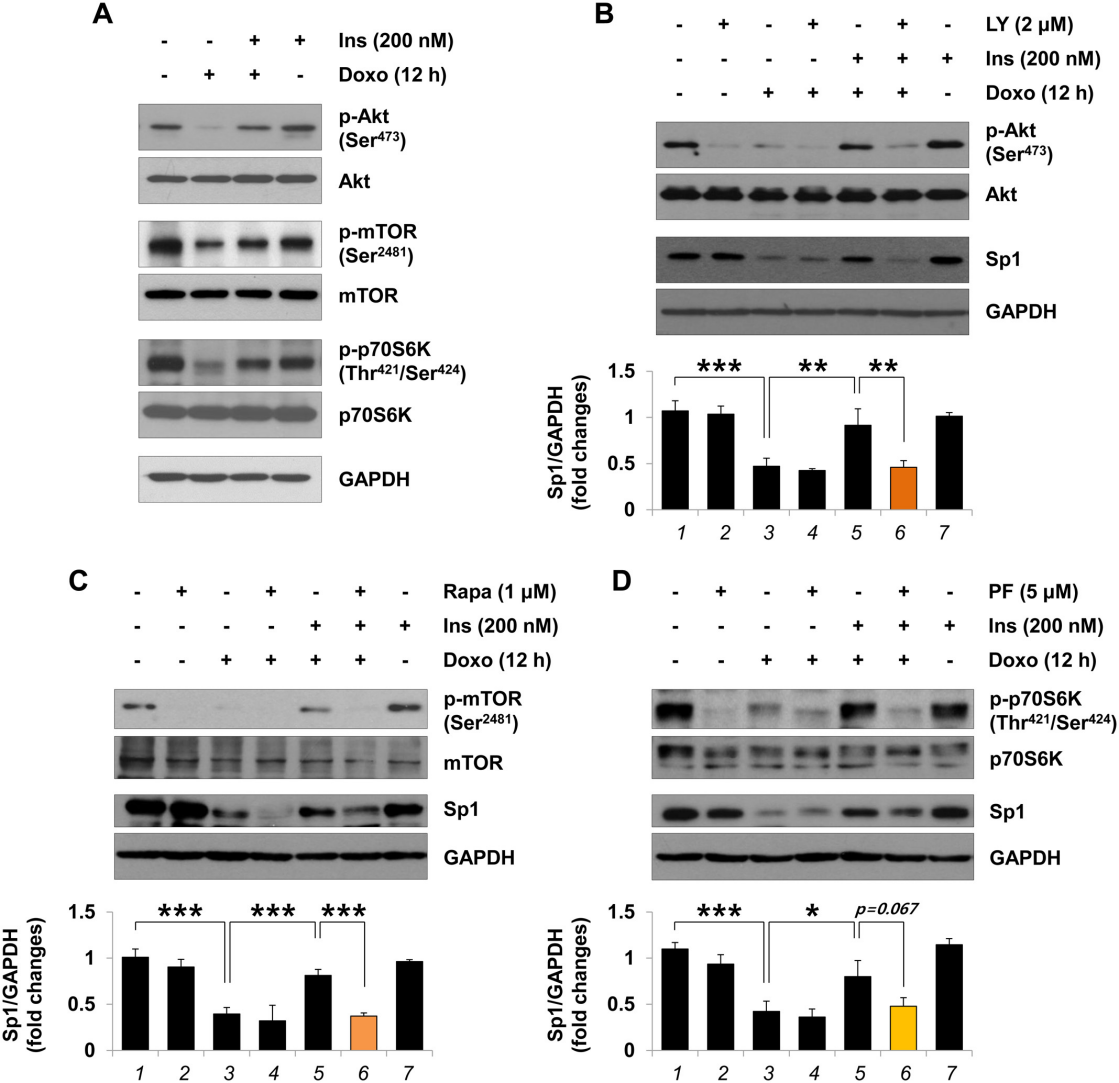
Inhibitory effect of insulin on the doxorubicin-induced Sp1 degradation via PI3K/Akt/mTORC1 pathway in H9c2 cardiac myocytes. (A) Serum-deprived H9c2 cardiac myocytes were left untreated or pretreated with 200 nM insulin (*Ins*) for 1 h and treated with 1 μM doxorubicin (*Doxo*) for 12 h. (B–D) Cells were pretreated with 2 μM PI3K inhibitor LY294002 (*LY*) (B), 1 μM mTORC1 inhibitor rapamycin (*Rapa*) (C), or 5 μM p70S6K inhibitor PF4708671 (*PF*). (D) for 1 h, followed by treatment with insulin (200 nM) for 1 h and then with doxorubicin (1 μM) for 12 h. Whole cell lysates were analyzed by immunoblotting for protein levels or phosphorylation status of Akt, mTORC1 and p70S6K and for protein levels of Sp1 using antibodies listed in Materials and Methods. Note that these blots represent one of three independent experiments. Graphs represent the mean ±S.D. of the normalized densitometric analyses of Sp1 protein levels (*p < 0.05; **p < 0.01; ***p < 0.001, n = 3).

Insulin-elicited cardiovascular protection is mediated by activation of PI3K/Akt/mTOR pathway [32–36]. First, we determined whether insulin signals to the PI3K/mTOR/p70S6K pathway to mediate cytoprotection against doxorubicin-mediated cell death. The results shown in Fig 6A domonstrated that doxorubicin treatment of H9c2 cardiac myocytes reduced the phosphorylation levels of Akt (downstream target of PI3K), mTORC1 and p70S6K. However, when the cells were pretreated with insulin, doxorubicin-mediated suppression of Akt, mTORC1 and p70S6K activation was perturbed. To determine the role of PI3K, mTOR and p70S6K in mediating the inhibitory effect of insulin on the Sp1 degradation stimulated by doxorubicin, three inhibitors were used; PI3K inhibitor LY294002, mTORC1 inhibitor rapamycin, and p70S6K inhibitor PF4708671. As shown in Fig 6B to 6D, insulin-stimulated phosphorylation of Akt, mTORC1, and p70S6K was inhibited by LY294002 (Fig 6B), rapamycin (Fig 6C), and PF4708671 (Fig 6C), respectively. Pretreatment with LY294002, rapamycin, and PF4708671 blocked the ability of insulin to inhibit doxorubicin-induced Sp1 degradation (Fig 6B to 6D, *lane 6*). It should be pointed out that pretreatment with LY294002, rapamycin, and PF4708671 did not affect total Akt, mTORC1, p70S6K or Sp1 protein levels (Fig 6B to 6D, *lane 2*).

### Insulin protects H9c2 cardiac myocytes from doxorubicin toxicity by Sp1-mediated transactivation of survivin via the PI3K/mTORC1/p70S6K pathway

**Fig 7:**
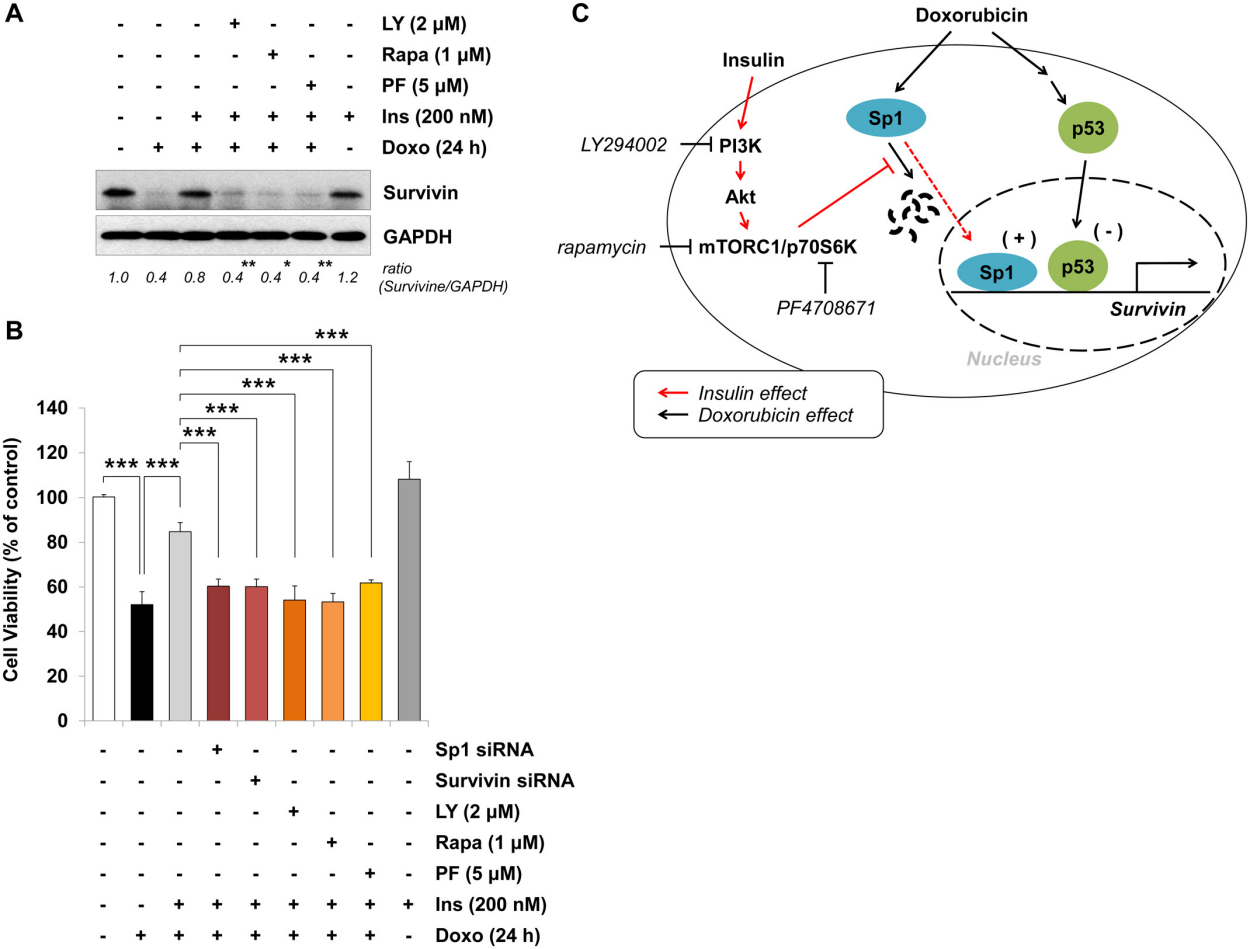
Survivin-mediated protective effect of insulin on the doxorubicin-induced cardiac cell death. After pretreatment with the indicated inhibitors for 1 h (A, B), or transfection with either survivin siRNA or Sp1 siRNA for 24 h (B), cells were left untreated or treated with insulin (*Ins*) for 1 h, followed by doxorubicin (*Doxo*) for 24 h. (A) Whole cell lysates were immunoblotted with anti-survivin antibodies. Numbers represent the mean ±S.D. of the normalized densitometric analyses of survivin protein levels (n = 4). (B) Cell viability was assessed by the MTT assay (n = 3 performed in triplicates). Values are mean ±S.D. (*p < 0.05; **p < 0.01; ***p < 0.001). (C) Schematic diagram of insulin-induced cardiac myocytes protection against doxorubicin toxicity. Pro-apoptotic signaling cascades induced by doxorubicin are shown as black lines and pro-survival (or anti-apoptotic) signaling pathways induced by insulin are shown as red lines. (+) indicates positive transcriptional regulator (e.g. activator); (-) indicates negative transcriptional regulator (e.g. repressor).

As shown in Fig 7A, pretreatment with LY294002, rapamycin, and PF4708671 also blocked the ability of insulin to inhibit doxorubicin-induced survivin down-regulation (*lane 4–6*). Finally, we investigated the effect of either survivin or Sp1 knockdown, or pharmacological inhibition of the PI3K/mTOR/p70S6K pathway on the insulin-induced protection of H9c2 cardiac myocytes. Consistent with the result shown in Figs 2E and 5D, doxorubicin treatment reduced cell viability and this was perturbed with insulin pretreatment. Interestingly, the viability of cells transfected with survivin siRNA (60.3 ± 3.2%) or Sp1 siRNA (60.1 ± 3.4%), or pretreated with PI3K inhibitor (54.1 ± 6.3%), mTORC1 inhibitor (53.3 ± 3.8%), or p70S6K inhibitor (61.8 ± 1.4%) prior to doxorubicin stimulation were similar to that of doxorubicin only-treated group (52.1 ± 5.8%) (Fig 7B). These findings demonstrated that insulin-stimulated activation of PI3K/mTORC1/p70S6K and expressions of Sp1 and survivin are required for insulin-mediated protection against doxorubicin-stimulated cell death of cardiac myocytes.

## Discussion

The major findings of this study are as follows: First, Sp1 was down-regulated following doxorubicin treatment, possibly by proteasome-mediated proteolysis. Insulin pretreatment blocked doxorubicin-mediated Sp1 degradation by activating the survival signaling cascade PI3K/Akt/mTORC1 pathway. In addition, reducing Sp1 expression with Sp1 knockdown successfully blunted the insulin-induced cytoprotection. Second, our ChIP assay revealed that insulin pretreatment perturbed doxorubicin-mediated inhibition of the association of the transcription factor Sp1 with the *survivin* gene promoter region (between -265 and -9). Conversely, insulin pretreatment perturbed doxorubicin-stimulated p53 expression and phosphorylation at Ser^15^. Consistently, our ChIP assay showed that doxorubicin-stimulated binding of p53 to the *survivin* gene promoter was completely suppressed by insulin pretreatment.

Since Altieri and his colleagues reported survivin as a novel anti-apoptosis gene expressed in cancer and lymphoma [37], the promoter region of *survivin* gene has been studied exclusively in various cancer cell lines, mostly originated from mouse or human [12, 38–40]. In this study, we investigated the regulation of *survivin* gene expression by Sp1 in mediating insulin-stimulated protection against doxorubicin-mediated toxicity of H9c2 cells, a rat-derived noncancerous myoblast cell line. Changes in gene expression of Sp1 following doxorubicin is controversial. In human colon carcinoma HCT116 cell line, Sp1 expression is not affected by doxorubicin treatment, however p53 protein is markedly increased in the same condition [13, 41]. Doxorubicin treatment up-regulates Sp1 protein and mRNA levels in the human breast adenocarcinoma MCF-7 [42] and chronic myeloid leukemic cell line K562 cells, respectively [43]. In contrast, similar to our results, doxorubicin induces Sp1 down-regulation in doxorubicin-resistant MCF-7 (MCF-7/Dox) cell line, whereas Sp1 mRNA is unchanged by doxorubicin treatment [44]. Although Sp1 is believed to be a transcriptional activator, its possible role as a transcriptional repressor in the *survivin* gene regulation also has been proposed. Inconsistent with our ChIP data, in which Sp1 acted as a transcriptional activator, p53 appears on the promoter without the loss of Sp1 to mediate dual function of both transcription activator and repressor after 9 h of doxorubicin treatment [13].

The increased level of p53 protein can be accomplished by enhancing protein stability instead of inducing transcription or translation. Our results supported the notion that DNA damage, e.g. doxorubicin stimuli, induces phosphorylation of p53 at Ser^15^ and leads to a reduced interaction between p53 and its negative regulator, the oncoprotein MDM2, which inhibits p53 accumulation by targeting it for ubiquitination and proteasomal degradation [45, 46]. Surprisingly, the increase in the phosphorylation of p53 at Ser^15^ was reduced in H9c2 cardiac myocytes that were pretreated with insulin. To better understand the mechanisms for cytoprotection mediated by insulin, more studies will be required to identify the specific kinases that are activated by doxorubicin to mediate the Ser^15^ phosphorylation and define the precise correlation between the post-translational modification (phosphorylation or acetylation) of p53 and its function as transcription factor [13, 47]. Chromatin modification in the promoter region may play a crucial role in the *survivin* gene silencing by p53 [6].

As shown in Fig 4, we designed an experiment using a potent proteasome inhibitor MG132 to inhibit doxorubicin-stimulated degradation of the Sp1 protein. Our findings are supported by a recent publication that showed MG132 pretreatment of MCF-7/Dox cells inhibits the doxorubicin-induced Sp1 down-regulation (the level of mRNA is not changed) [44]. Moreover, inhibitor studies shown in Fig 6 demonstrated a connection between the mTOR pathway and proteasome-mediated protein degradation [48]. We cannot exclude the possibility that another signaling pathway might be involved in the insulin-induced inhibition of Sp1 degradation. As shown in Fig 6, the efficiencies of the three inhibitors (LY294002 > rapamycin > PF4708671) suggested that a pathway might share the upstream kinases, PI3K, Akt and mTORC1, but they might have distinct downstream mediators. Insulin-like growth factor-I (IGF-I) induces the expression of survivin by increasing the translation of mRNA through mTOR-dependent p70S6K activation, rather than by regulating gene transcription or protein stability [28, 49]. In a more complicated study by Song et al., IGF-I reverses suppression of *survivin* gene expression by TGF-β [49], whose *survivin* gene down-regulation depends on two cell cycle repressor elements in the *survivin* promoter region, a cell cycle-dependent element (CDE) and a cell cycle genes homology region (CHR); and two transcription factors Smad2 and Smad3 [50]. Although it has been suggested that survivin plays a critical role in the cardioprotective effect of insulin through the activation of a survival cascade [30], the mechanism how insulin-elicited activation of the PI3K/mTORC1/p70S6K pathway regulates *survivin* gene transcription remains to be elucidated. In this study, it was successfully confirmed by ChIP assays that insulin could reverse the doxorubicin-induced survivin down-regulation by maintaining the positive regulator Sp1 [51] on the promoter of *survivin* gene and removing the negative regulator p53 [6] from the promoter of *survivin* gene.

Clinically, the doxorubicin-induced cardiotoxicity is one of the most important sequelae of current chemotherapy against various cancers. A safe and effective method for reducing anthracyclin-induced cardiotoxicity remains elusive [52]. In this study, we tested whether the physiologic concentration of insulin could limit the doxorubicin-induced cardiotoxicity. Recently, insulin-like growth factor (IGF) and its binding protein (IGFBP) involve in cancer development, progression and anti-cancer drug resistance, thus IGF/IGFBP is an emerging target of cancer therapy [53, 54]. In line with these findings, insulin could be harmful in cancer patients. Additionally, insulin may be beneficial by reducing host nutritional toxicity and result in improvement of antitumor efficacy [55]. Therefore, further research should be warranted to assess the clinical feasibility of insulin in cancer patients.

Taken together, our results support the hypothesis (a simplified scheme depicted in Fig 7C) that *i)* insulin prevents Sp1 from the doxorubicin-induced proteasomal degradation via the PI3K/mTORC1/p70S6K pathway; *ii)* the preserved Sp1 level by insulin transactivates the *survivin* gene by direct binding to its promoter region; and *iii)* survivin mediates protective effect of insulin against doxorubicin toxicity.

## Supporting Information

S1 Fig Effect of insulin on release of Smac/DIABLO and Bcl-2 family. (A, B) Serum-deprived cells were left untreated or pretreated with insulin (200 nM) for 1 h and treated with doxorubicin (1 μM) for 24 h. (A) Mitochondrial (*Mito*) and cytosolic (*Cyto*) fractions were separated by SDS-PAGE gel and analyzed by immunoblotting with anti-Smac/DIABLO antibodies. (B) Whole cell lysates were immunoblotted with anti-Bcl-2, anti-BAX and anti-caspase-3 (active form) antibodies. VDAC1 and β-actin are used as a loading control for mitochondrial and cytosolic fractions, respectively. Note that blots represent one of three independent experiments.
(PDF)

S2 Fig Effect of doxorubicin and/or insulin on survivin expression at protein levels in H9c2 cardiac myocytes. Eight replicates of Western blot for survivin. Whole cell lysates were separated by SDS-PAGE gel and analyzed by immunoblotting with antibodies against survivin and GAPDH.
(PDF)

S3 Fig Effect of doxorubicin on survivin expression at both protein and mRNA levels in H9c2 cardiac myocytes. (A, B) H9c2 cardiac myocytes were treated with doxorubicin for the indicated time points. Whole cell lysates were separated by SDS-PAGE gel and analyzed by immunoblotting with antibodies against p-p53, p53, Sp1, survivin and GAPDH (A), and total RNA was analyzed by RT-PCR (28 cycles) using primers specific to *survivin* and *GAPDH* gene (B).
(PDF)

S4 Fig Effect of doxorubicin and/or insulin on Sp1 mRNA expression in H9c2 cardiac myocytes. (A) H9c2 cardiac myocytes were treated with the increased concentration of doxorubicin (*Doxo*) up to 1 μM for 12 h, and (B) cells were pretreated with insulin (200 nM) for 1 h and treated with doxorubicin (1 μM) for 12 h. Sp1 mRNA amount was determined by semi-qPCR (22–30 cycles).
(PDF)
