# Expression of Concern: Insulin Protects Cardiac Myocytes from Doxorubicin Toxicity by Sp1-Mediated Transactivation of Survivin

**DOI:** 10.1371/journal.pone.0285865

**Published:** 2023-05-11

**Authors:** The PLOS ONE Editors

The corresponding author requested retraction of this article [1] and stated that this request followed from an investigation of concerns about the inclusion of Sung Ku Kang in the article’s author list. However, PLOS has not received the documentation needed to verify the concerns and clarify whether retraction is warranted per the Committee on Publication Ethics (COPE) Retraction Guidelines [2] and PLOS policies.

In reviewing this matter, PLOS noted that the primary data were not provided with the published article [1], contrary to the Data Availability Statement. The authors did not respond to requests for the primary data or queries about whether the data are still available, and so the article [1] does not currently comply with the PLOS Data Availability policy.

The *PLOS ONE* Editors issue this Expression of Concern to notify readers of the above issues which are unresolved as of the time of this notice.
