# Supplementary figures and images for: Insulin Protects Cardiac Myocytes from Doxorubicin Toxicity by Sp1-Mediated Transactivation of Survivin
Source: PLoS One. 2015 Aug 13;10(8):e0135438. doi: 10.1371/journal.pone.0135438 (PMC4535909; doi:10.1371/journal.pone.0135438)

**A**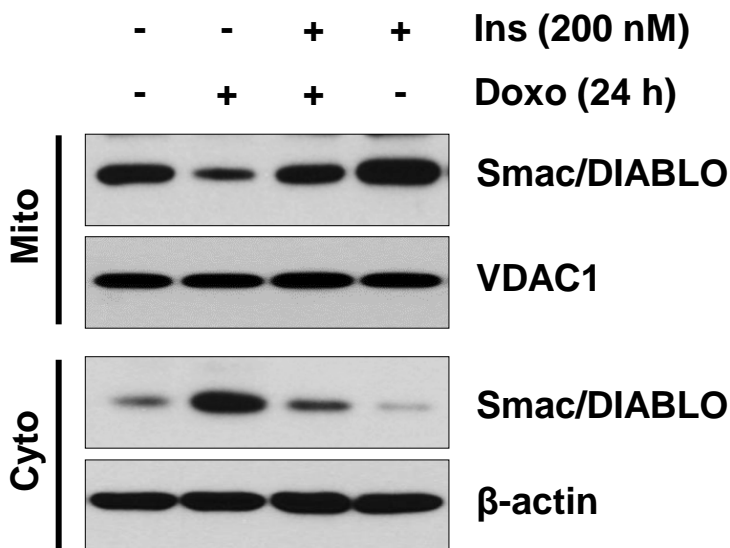**B**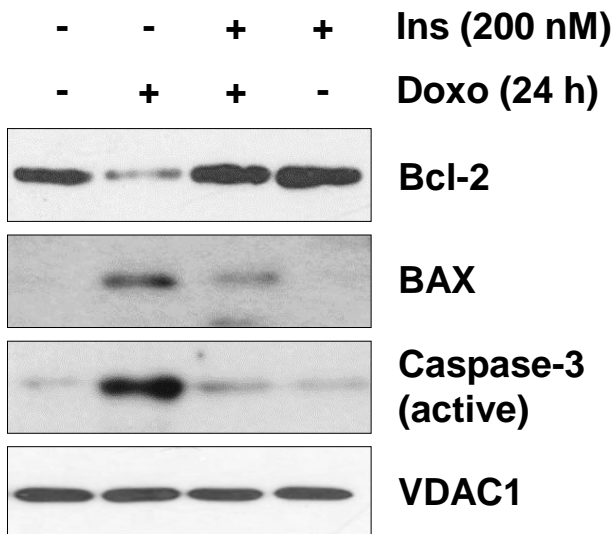

Supplement: S1 Fig — (A, B) Serum-deprived cells were left untreated or pretreated with insulin (200 nM) for 1 h and treated with doxorubicin (1 μM) for 24 h. (A) Mitochondrial (Mito) and cytosolic (Cyto) fractions were separated by SDS-PAGE gel and analyzed by immunoblotting with anti-Smac/DIABLO antibodies. (B) Whole cell lysates were immunoblotted with anti-Bcl-2, anti-BAX and anti-caspase-3 (active form) antibodies. VDAC1 and β-actin are used as a loading control for mitochondrial and cytosolic fractions, respectively. Note that blots represent one of three independent experiments. (PDF) [file pone.0135438.s001.pdf]

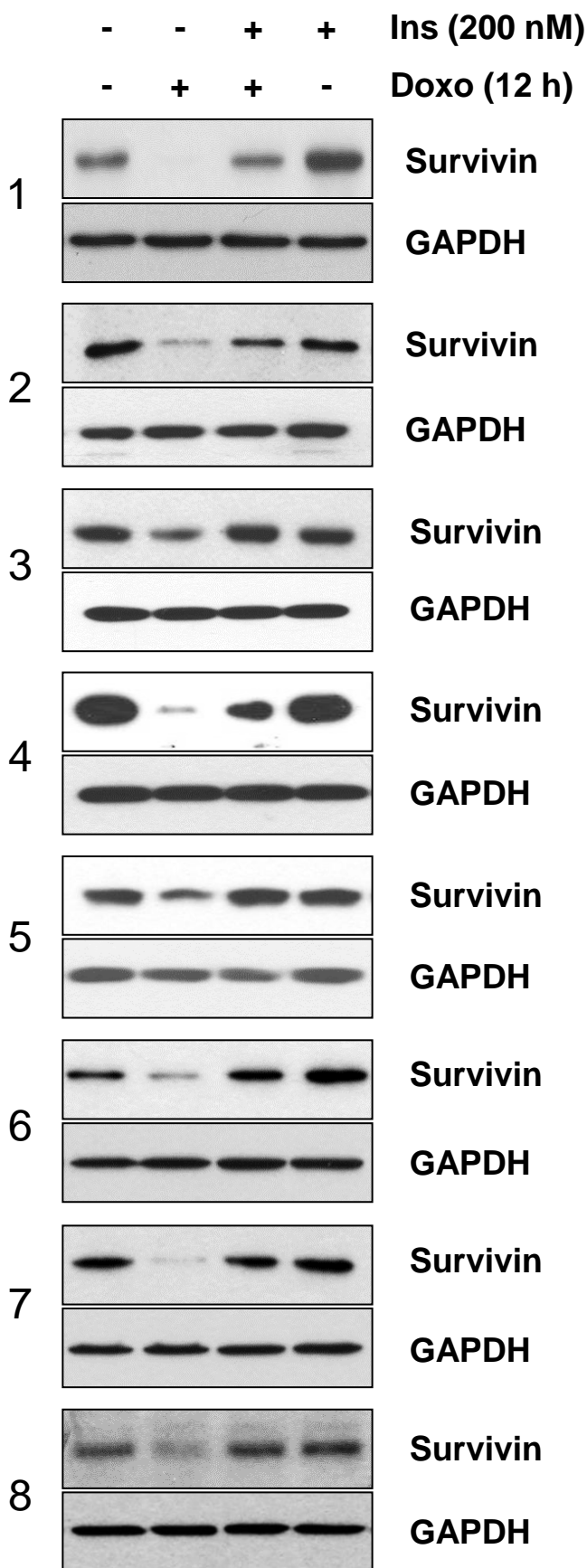

Supplement: S2 Fig — Eight replicates of Western blot for survivin. Whole cell lysates were separated by SDS-PAGE gel and analyzed by immunoblotting with antibodies against survivin and GAPDH. (PDF) [file pone.0135438.s002.pdf]

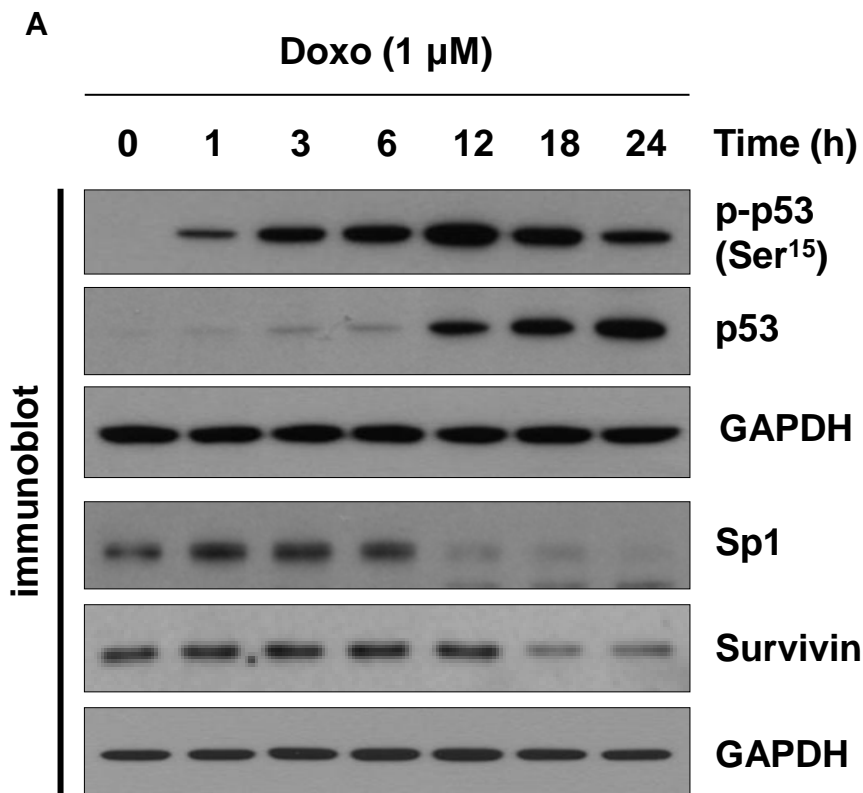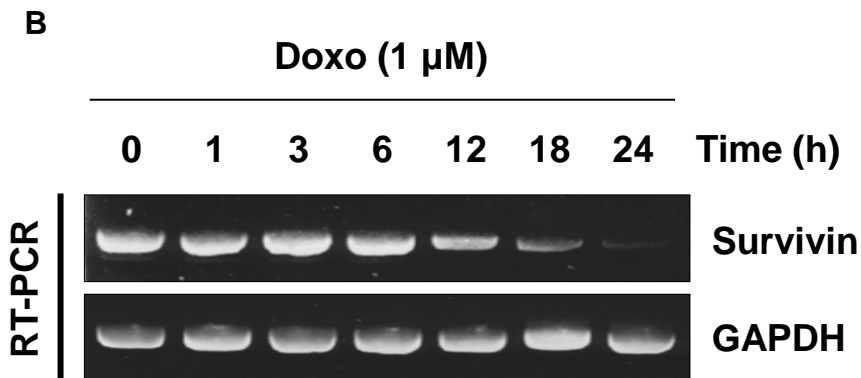

Supplement: S3 Fig — (A, B) H9c2 cardiac myocytes were treated with doxorubicin for the indicated time points. Whole cell lysates were separated by SDS-PAGE gel and analyzed by immunoblotting with antibodies against p-p53, p53, Sp1, survivin and GAPDH (A), and total RNA was analyzed by RT-PCR (28 cycles) using primers specific to survivin and GAPDH gene (B). (PDF) [file pone.0135438.s003.pdf]

**A**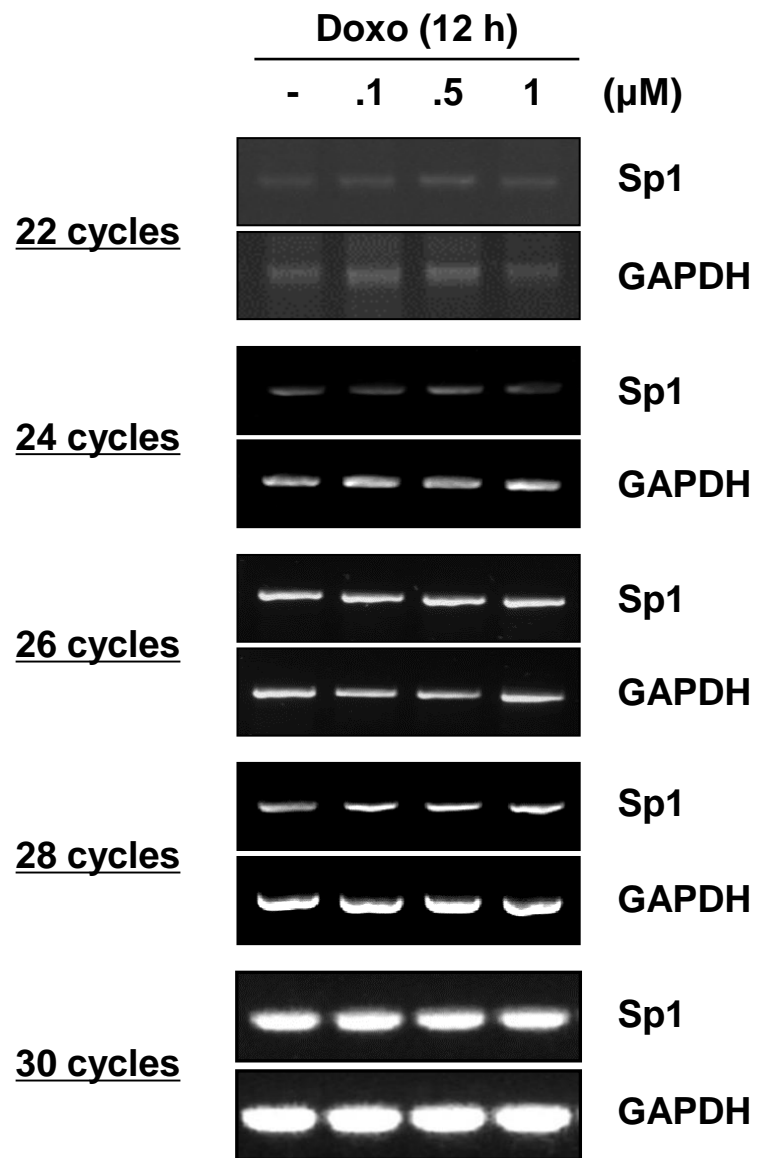**B**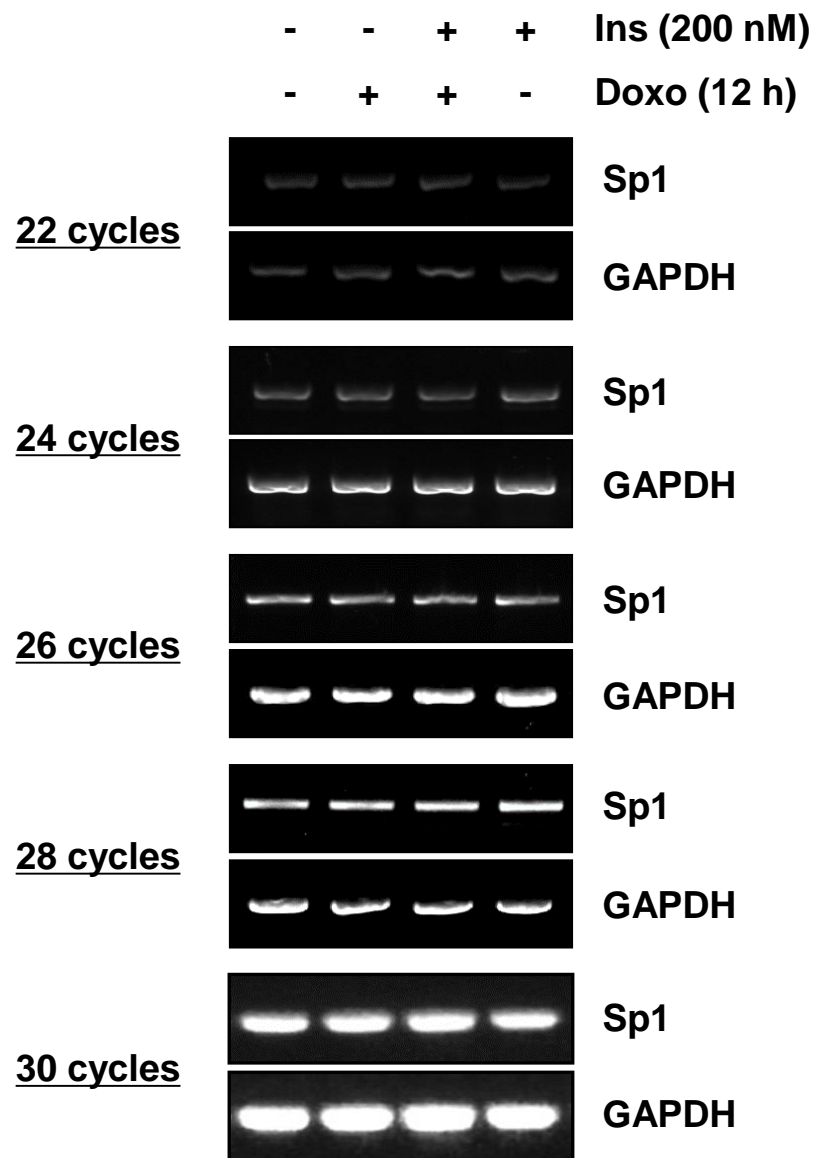

Supplement: S4 Fig — (A) H9c2 cardiac myocytes were treated with the increased concentration of doxorubicin (Doxo) up to 1 μM for 12 h, and (B) cells were pretreated with insulin (200 nM) for 1 h and treated with doxorubicin (1 μM) for 12 h. Sp1 mRNA amount was determined by semi-qPCR (22–30 cycles). (PDF) [file pone.0135438.s004.pdf]
